# Paeonol Protects Against Methotrexate-Induced Nephrotoxicity *via* Upregulation of P-gp Expression and Inhibition of TLR4/NF-κB Pathway

**DOI:** 10.3389/fphar.2022.774387

**Published:** 2022-02-04

**Authors:** Mohamed A. Morsy, Azza A. K. El-Sheikh, Sara Mohamed Naguib Abdel-Hafez, Mahmoud Kandeel, Seham A. Abdel-Gaber

**Affiliations:** ^1^ Department of Pharmaceutical Sciences, College of Clinical Pharmacy, King Faisal University, Al-Ahsa, Saudi Arabia; ^2^ Department of Pharmacology, Faculty of Medicine, Minia University, Minia, Egypt; ^3^ Department of Basic Sciences, College of Medicine, Princess Nourah Bint Abdulrahman University, Riyadh, Saudi Arabia; ^4^ Department of Histology and Cell Biology, Faculty of Medicine, Minia University, Minia, Egypt; ^5^ Department of Biomedical Sciences, College of Veterinary Medicine, King Faisal University, Al-Ahsa, Saudi Arabia; ^6^ Department of Pharmacology, Faculty of Veterinary Medicine, Kafrelsheikh University, Kafrelsheikh, Egypt

**Keywords:** methotrexate, paeonol, P-glycoprotein, nephrotoxicity, KIM-1, TLR4, NF-κB, IL-1β

## Abstract

Methotrexate (MTX) is a well-known anticancer drug that causes nephrotoxicity as a side effect. To investigate the mechanisms by which paeonol, a natural phenolic compound, can protect against MTX-induced nephrotoxicity, paeonol (100 mg/kg/day orally) was given to rats for 10 days, with or without MTX (20 mg/kg once i.p. at day 5). Compared to control, MTX caused nephrotoxic effects manifested by increased serum urea and creatinine and distortion in renal histological architecture, with a significant increase in the mean glomerular diameter and upregulation of kidney injury molecule-1. MTX caused oxidative stress manifested by decreasing reduced glutathione and superoxide dismutase while increasing malondialdehyde and nitric oxide. MTX also induced renal inflammation by upregulating TLR4, NF-κB, and IL-1β and caused apoptosis by induction of caspase 3. Administering paeonol with MTX improved kidney functional and structural parameters, as well as all oxidative, inflammatory, and apoptotic markers tested. Interestingly, both MTX and paeonol increased the expression of the renal efflux transporter P-glycoprotein (P-gp) that helps in MTX elimination, and their drug combination further upregulated renal P-gp. *In silico*, paeonol was neither a substrate nor an inhibitor of P-gp, suggesting that its effect on P-gp is not on functional but on the expression level. *In vitro*, paeonol and MTX were administered to colon cancer cells and their combination caused a progressive cellular cytotoxic effect, which was dose-dependent with the increase of paeonol concentration. In conclusion, paeonol protects against MTX-induced nephrotoxicity through antioxidant, anti-inflammatory, and antiapoptotic mechanisms and might potentiate MTX chemotherapeutic efficacy.

## Introduction

Methotrexate (MTX) is an antifolate that has been highly used in large doses in the treatment of a wide variety of cancers (Hannoodee and Mittal, 2021). Unfortunately, such a high dose of MTX may be accompanied by multi-organ damage, including the kidneys. Since MTX depends mainly on renal elimination for excretion, MTX-induced kidney damage may cause accumulation of MTX that would further worsen its systemic hazards (Widemann and Adamson, 2006), causing a vicious circle. The mechanisms involved in MTX-induced nephrotoxicity may involve the accumulation of MTX in the kidney causing oxidative stress and free radical formation that might trigger an inflammatory process, ultimately leading to programmed cell death—apoptosis (El-Sheikh et al., 2015).

One of the main factors affecting MTX renal accumulation is the number of efflux transporters situated at the apical membrane of the proximal tubules that actively pumps MTX into the urine, including breast cancer resistance protein (BCRP) and multidrug resistance proteins (MRPs) MRP2 and MRP4, as well as P-glycoprotein (P-gp). P-gp, a member of the ATP-binding cassette subfamily B member 1 (ABCB1), is one of these efflux transporters that accepts MTX as its substrate (de Graaf et al., 1996). Unfortunately, P-gp is also expressed in a large number of human cancers (Breier et al., 2005), where it confers multidrug resistance against chemotherapeutic agents, including MTX. Thus, trying to add a nephroprotective adjuvant with MTX regimens would be a double-edged tool, on the one hand, attempting to ameliorate MTX-induced toxicity, and on the other hand, trying not to tamper with MTX anticancer efficacy.

Paeonol is a natural phenolic compound present in the root bark of *Paeonia suffruticosa* and was reported by previous studies to modulate oxidative, inflammatory, and apoptotic pathways, as well as has antitumor properties (Zhang et al., 2019; Vellasamy et al., 2021). Few *in vitro* studies have shown that paeonol might modulate the expression of efflux transporters that accept MTX as a substrate, such as BCRP and P-gp (Zhang et al., 2015). One recent *in vivo* study reported that paeonol could affect testicular expression levels of P-gp (Morsy et al., 2020a). Paeonol seems to act as an “adaptogen.” For example, paeonol was reported to protect normal body cells against apoptosis (Liu et al., 2021; Tang et al., 2021), whereas it can induce apoptosis in malignant cells (Li et al., 2019; Liu et al., 2020). For such unique properties, in the current study, the possible protective effect of paeonol on renal toxic effects of MTX was tested, and the mechanisms involved were explored *in vivo*, focusing on the role of P-gp. In addition, the effect of paeonol on MTX-induced cytotoxicity was tested on cancer cells *in vitro*.

## Materials and Methods

### Drugs and Chemicals

Paeonol was purchased from Sigma-Aldrich (St. Louis, MO, United States), and MTX was obtained from Minapharm Pharmaceuticals (Cairo, Egypt). Ready-to-use kidney injury molecule-1 (KIM-1; PA5-79345), nuclear factor-κB p65 subunit (NF-κB/p65; PA5-17264), and cleaved caspase 3 (PA5-23921) rabbit polyclonal antibodies were procured from Thermo Fisher Scientific (Waltham, MA, United States), while P-gp mouse monoclonal antibodies (sc-390883) were brought from Santa Cruz Biotechnology (Dallas, TX, United States). Other chemicals used were of analytical grade and were brought from their commercial sources.

### Animals and Experimental Design

Male Wistar rats weighing 180–220 g were acquired from the National Research Center (Giza, Egypt) and were caged under standard housing conditions (25°C ± 1 and 12-h light/dark cycle). Rats were offered ordinary chow and water ad libitum. The study protocol was performed in accordance with research ethical standards of the Faculty of Medicine-Research Ethics Committee, Minia University, Egypt (ethical approval No. 630/6/2020), which is consistent with EU directive 2010/63/EU. After a week of acclimatization, 24 rats were divided into four groups (*n* = 6). The first group served as the untreated control. Paeonol, suspended in 0.5% carboxymethyl cellulose solution, was given to the second group as a single daily oral dose of 100 mg/kg/day for 10 days (Al-Taher et al., 2020). The third group received MTX alone as a single i.p. dose of 20 mg/kg MTX (Morsy et al., 2020a) on the fifth day of the experiment, while the fourth group received a combination of MTX/paeonol regimen.

### Sample Collection

At the end of the 10th day, rat blood samples were collected and centrifuged at 5,000 rpm for 15 min for sera collection. Rats were euthanized, and their kidneys were excised. A longitudinal slice of one of each rat’s kidneys was fixed in 10% neutral-buffered formalin and embedded in paraffin, from which sections of 5 μm thickness were cut out and mounted on glass slides for histopathological and immunohistochemical staining. The rest of the kidneys were homogenized in 10% w/v ice-cold, 0.01 M, pH 7.4 phosphate buffer. After centrifugation of the homogenate at 4,000 rpm for 15 min, the supernatant of kidney homogenate was kept at −80°C until used for renal tissue biochemical measurements and real-time polymerase chain reaction (PCR).

### Biochemical Studies

Serum urea and creatinine as well as renal tissue reduced glutathione (GSH) were determined using commercially available kits (Biodiagnostic, Giza, Egypt). Renal superoxide dismutase (SOD) activity was evaluated *via* a method based on SOD inhibition of pyrogallol autoxidation (Marklund and Marklund, 1974). Briefly, kidney homogenates were mixed with Tris–HCl (pH 8.2) and pyrogallol (15 mM), and the absorbance of the sample was measured against blank at 420 nm over 3 min. The findings were presented as U/g tissue. The major lipid peroxidation product malondialdehyde (MDA) was measured as MDA–thiobarbituric acid adduct formed under high temperature and acidic conditions using 1,1,3,3-tetramethoxypropane as a standard. The findings were presented as nmol/g tissue (Buege and Aust, 1978). For the determination of nitric oxide (NO), the Griess method was used for estimation of the stable oxidation end products of NO (nitrites and nitrates), where the latter is reduced to the former by copperized cadmium granules, and the color change is revealed by the Griess reagent in an acidic medium. This color is then measured spectrophotometrically at 540 nm, and the findings are presented in nmol/g tissue (Sastry et al., 2002).

### Histopathological and Immunohistochemical Staining

The renal tissue sections were stained with the hematoxylin and eosin (H&E) stain and examined under a light microscope (Olympus CX23LEDRFS1, Olympus, Tokyo, Japan). Morphometric estimation was performed using Leica QWin 500 image analysis software (Leica Microsystems, Wetzlar, Germany) for evaluation of the mean diameter of Malpighian renal corpuscles per section (Beshay et al., 2020). Other sections were stained with the periodic acid–Schiff (PAS) stain for examining the integrity of the glomerular membrane. For an immunohistochemical analysis, slides were stained with ready-to-use rabbit polyclonal primary antibodies of KIM-1, NF-κB/p65, and cleaved caspase 3 as well as P-gp, according to the manufacturer’s protocol. The mean area fraction of PAS staining, as well as that of immunohistochemical stains, was calculated *via* applying 10 non-overlapping fields for each group using ImageJ (freeware; rsbweb.nih.gov/ij).

### Determination of Renal TLR4 and IL-1β

For determination of renal toll-like receptor 4 (TLR4) and interleukin (IL)-1β mRNA, total RNA was extracted from kidney tissue homogenate by the RiboZol Reagent (AMRESCO, Solon, OH, United States), as instructed by the manufacturer. The primer pairs used for TLR4 were 5′-AAT​CCC​TGC​ATA​GAG​GTA​CTT​CCT​AAT-3' as the forward primer and 5′-CTC​AGA​TCT​AGG​TTC​TTG​GTT​GAA​TAA​G-3′ as the reverse primer (Al-Taher et al., 2020). For IL-1β, the forward primer was 5'-GTC​GTT​GCT​TGT​CTC​TCC​TTG​TA-3′ and the reverse primer was 5'-CAC​CTT​CTT​TTC​CTT​CAT​CTT​TG-3' (Leger et al., 2019). Applying 500 pg of RNA template per reaction, real-time PCR (Applied Biosystems 7500 Fast Real-Time PCR System, Foster City, CA, United States) was performed using a SensiFAST™ SYBR® Hi-ROX One-Step Kit (Bioline/Meridian Bioscience, Cincinnati, OH, United States) utilizing 70 nM of specific primers in 25 μL reaction volume. The data were quantified relative to glyceraldehyde 3-phosphate dehydrogenase (GAPDH) as a control gene (the forward primer was 5'-GTC​GGT​GTG​AAC​GGA​TTT​G-3′ and the reverse primer was 5'-CTT​GCC​GTG​GGT​AGA​GTC​AT-3′). The relative expression level of the studied genes was calculated using the comparative cycle threshold method (VanGuilder et al., 2008).

### *In Silico* P-gp Specificity

The P-gp specificity module (version 2016) of Percepta software (ACD/Labs, Toronto, Ontario, Canada) was used to predict P-gp specificity (P-gp substrates and/or inhibitors) from the chemical structure using a huge screening library. The Paeonol structure was drawn using ChemDraw software version 15 (PerkinElmer, Waltham, MA, United States).

### Cytotoxicity Assay

Cellular cytotoxicity was performed by applying 3-(4,5-dimethylthiazol-2-yl)-2,5-diphenyltetrazolium bromide (MTT) on HCT-116 colon cancer cells *in vitro*, as previously described (Morsy et al., 2020b). Briefly, HCT-116 colon cancer cells were seeded in 96-well plates for 24-h incubation in DMEM at a concentration of 10^4^ cells/well. Cells were then incubated for 24 h with paeonol at concentrations of 0.01, 0.1, 1, 10, or 100 µM and with MTX at concentrations of 0.1, 1, or 10 µM. After which, 15 μL MTT reagent (5 mg/mL phosphate-buffered saline) was supplemented, followed by incubation for 4 h at 37°C in the dark. After that, 100 μL of dimethyl sulfoxide at 37°C was added to each well to dissolve formazan crystals. The color change was then estimated at 540 nm using a microplate reader.

### Statistical Analysis

The data were analyzed using GraphPad Prism software, version 6.01 for Windows (San Diego, CA, United States) and were presented as mean ± SEM. Statistical analysis was performed by one one-way analysis of variance (ANOVA) followed by Tukey’s post hoc analysis test for multiple comparisons. For MTT cytotoxicity studies, a non-linear regression analysis was performed. The findings were considered significant if *p* was less than 0.05.

## Results

### Effect of Paeonol on Kidney Function and Oxidative Stress Parameters in MTX-induced Toxicity

At the end of the study, the levels of urea and creatinine were evaluated, and it showed that paeonol alone did not affect these kidney functional parameters, whereas MTX alone caused a significant increase in their levels compared to the control. Combined treatment with paeonol and MTX significantly prevented the effect compared to MTX alone. Similarly, paeonol alone had no significant effect on all tested oxidative stress markers, whereas MTX significantly decreased GSH and SOD while increasing MDA and NO levels. The combined paeonol/MTX-treated group however showed a significant increase in GSH and SOD as well as a significant decrease in MDA and NO compared to the group treated with MTX alone (Table 1).

**TABLE 1:** Effect of paeonol on serum urea and creatinine, and renal oxidative stress markers in methotrexate (MTX)-treated rats.

	Control	Paeonol	MTX	MTX/paeonol
Urea (mg/dl)	22.8 ± 1.5	23.8 ± 1.8	114.6 ± 7.2^a^	23.1 ± 1.9^b^
Creatinine (mg/dl)	0.78 ± 0.03	0.75 ± 0.04	1.71 ± 0.14^a^	0.81 ± 0.03^b^
GSH (nmol/g tissue)	634 ± 84	643 ± 39	271 ± 24^a^	681 ± 43^b^
SOD (U/g tissue)	6,039 ± 368	5,967 ± 594	505 ± 50^a^	8,153 ± 731^a,b^
MDA (nmol/g tissue)	23.9 ± 1.9	22.8 ± 1.8	56.4 ± 2.9^a^	26.4 ± 1.8^b^
NO (nmol/g tissue)	797 ± 74	822 ± 67	1,542 ± 37^a^	575 ± 52^b^

GSH: reduced glutathione, SOD: superoxide dismutase, MDA: malondialdehyde, NO: nitric oxide. Results show mean of six observations ± SEM. Values are considered significantly different when *p* < .05. ^a,b^ Significant difference compared to control or MTX, groups, respectively.

### Effect of Paeonol on Renal Histological Structure and Kidney Injury in MTX-induced Toxicity

**FIGURE 1:**
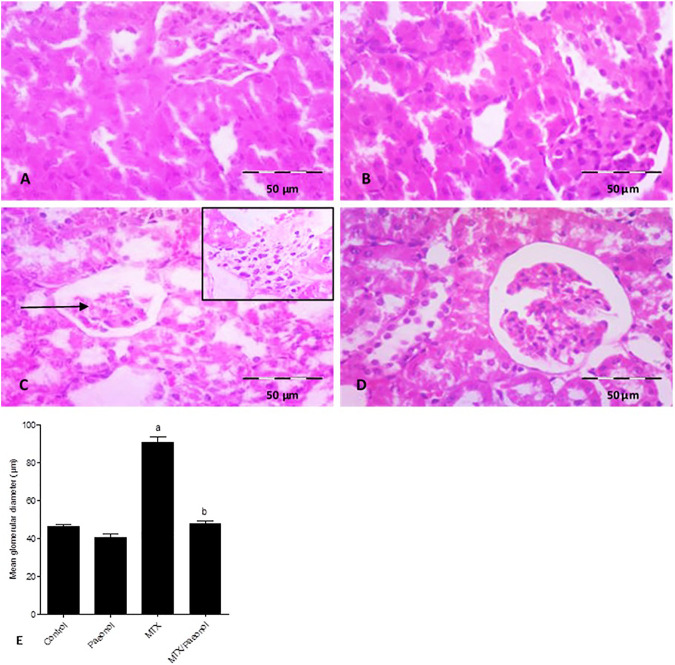
Histopathological picture of the kidney after methotrexate (MTX) and/or paeonol administration stained by hematoxylin and eosin (H and E). A photomicrograph of the rat kidney (×100) from **(A)** control, **(B)** paeonol-treated, **(C)** MTX-treated, and **(D)** MTX/paeonol-treated rats. Black arrow shows shrunken renal corpuscle, and the insert shows cellular infiltration. **(E)** Mean glomerular diameter obtained from sections of each animal (*n* = 6 for each group), six fields/section, where results were considered significantly different when *p* < .05. ^a,b^ Significant difference compared to control or MTX groups, respectively.

Histological examination of the kidney, stained by H and E, showed a normal renal structure in both control and paeonol-treated groups; the Malpighian renal corpuscles contained glomeruli surrounded with Bowman’s space, which is clear of any cell debris. The parietal layer of Bowman’s capsule is lined by flat squamous cells. Proximal convoluted tubules appeared with narrow lumina, rounded basal vesicular nuclei, and apical clear brush borders, while distal convoluted tubules appeared with wider lumina, central rounded nuclei, and unclear brush borders (Figures 1A,B, respectively). On the other hand, the MTX-treated group displayed shrunken renal corpuscles leaving wide Bowman’s spaces. The renal tubules appeared dilated with intraluminal cellular debris. The proximal tubules were seen with an almost lost brush border and showed cytoplasmic vacuolations. The dilated congested blood vessels and the focal areas of inflammatory cell infiltration surrounding thick arterioles were also detected (Figure 1C). Animals treated with both paeonol and MTX showed normal Malpighian renal corpuscles containing apparently normal glomerulus and Bowman’s spaces. Most proximal convoluted tubules had regained their brush borders. Still, vacuolations were seen in a few tubular cells (Figure 1D). The structural changes in the renal histological picture were reflected morphometrically in the mean glomerular diameter (Figure 1E), where paeonol treatment had no effect, while MTX treatment caused nearly a 2-fold increase in the mean glomerular diameter compared to control. Co-administration of paeonol and MTX significantly decreased the mean glomerular diameter compared to MTX alone.

**FIGURE 2:**
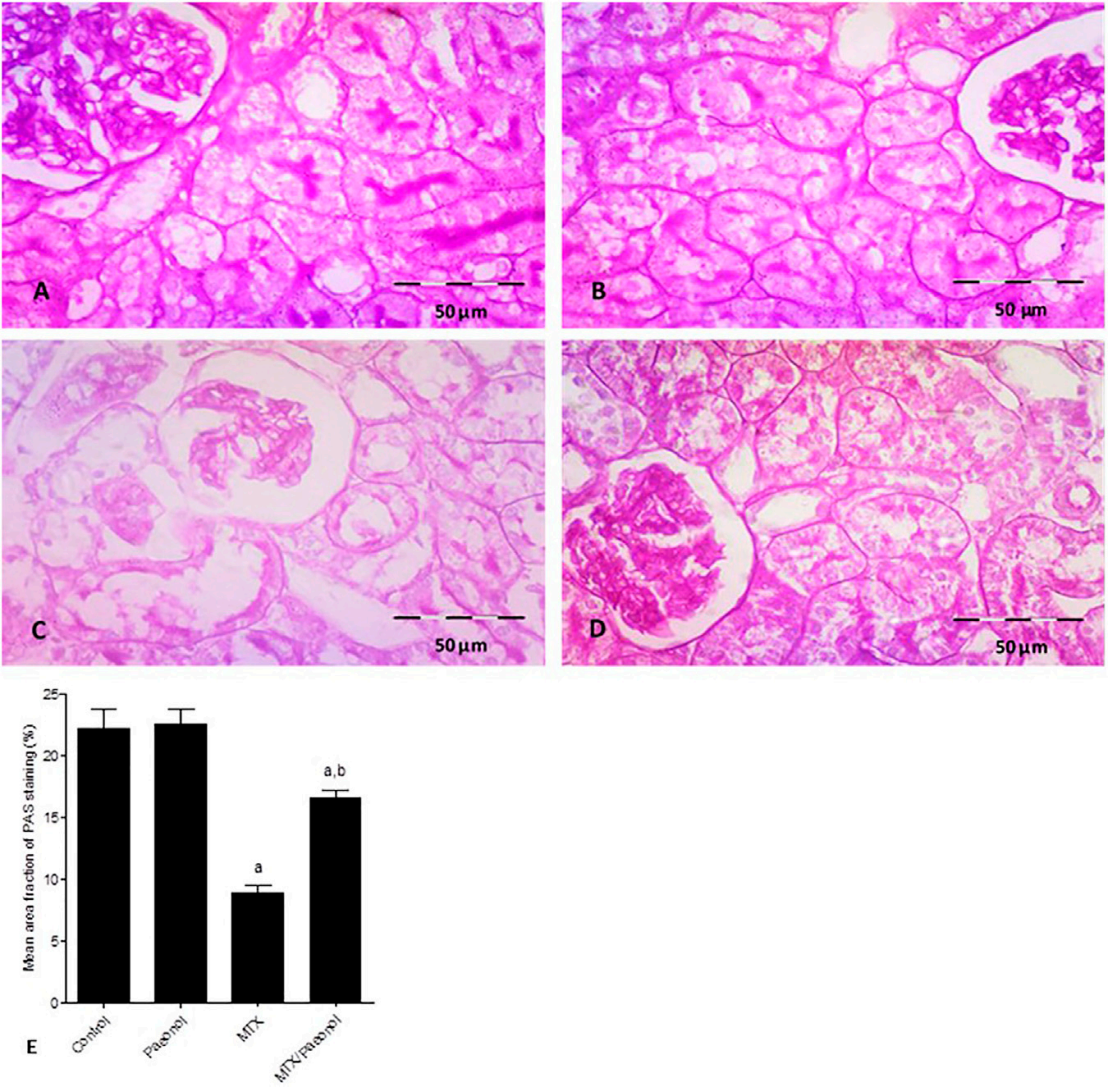
Histopathological picture of the kidney after methotrexate (MTX) and/or paeonol administration stained by periodic acid–Schiff (PAS). A photomicrograph of the rat kidney (×400) obtained from **(A)** control, **(B)** paeonol-treated, **(C)** MTX-treated, and **(D)** MTX/paeonol-treated rats. **(E)** Percent of mean area fraction of PAS staining obtained from sections of each animal (*n* = 6 for each group), six fields/section, where results were considered significantly different when *p* < .05. ^a,b^ Significant difference compared to control or MTX groups, respectively.

Using the PAS stain to highlight renal basement membranes showed that the control group had well-defined cell membranes (Figure 2A). Treatment with paeonol alone had no effect (Figure 2B), while MTX treatment caused significantly severe deterioration of the PAS-stained basement membrane (Figure 2C). Paeonol/MTX combined treatments significantly restored the integrity of the basement membrane (Figure 2D), as indicated with the analysis of the mean area fraction of PAS staining (Figure 2E).

**FIGURE 3:**
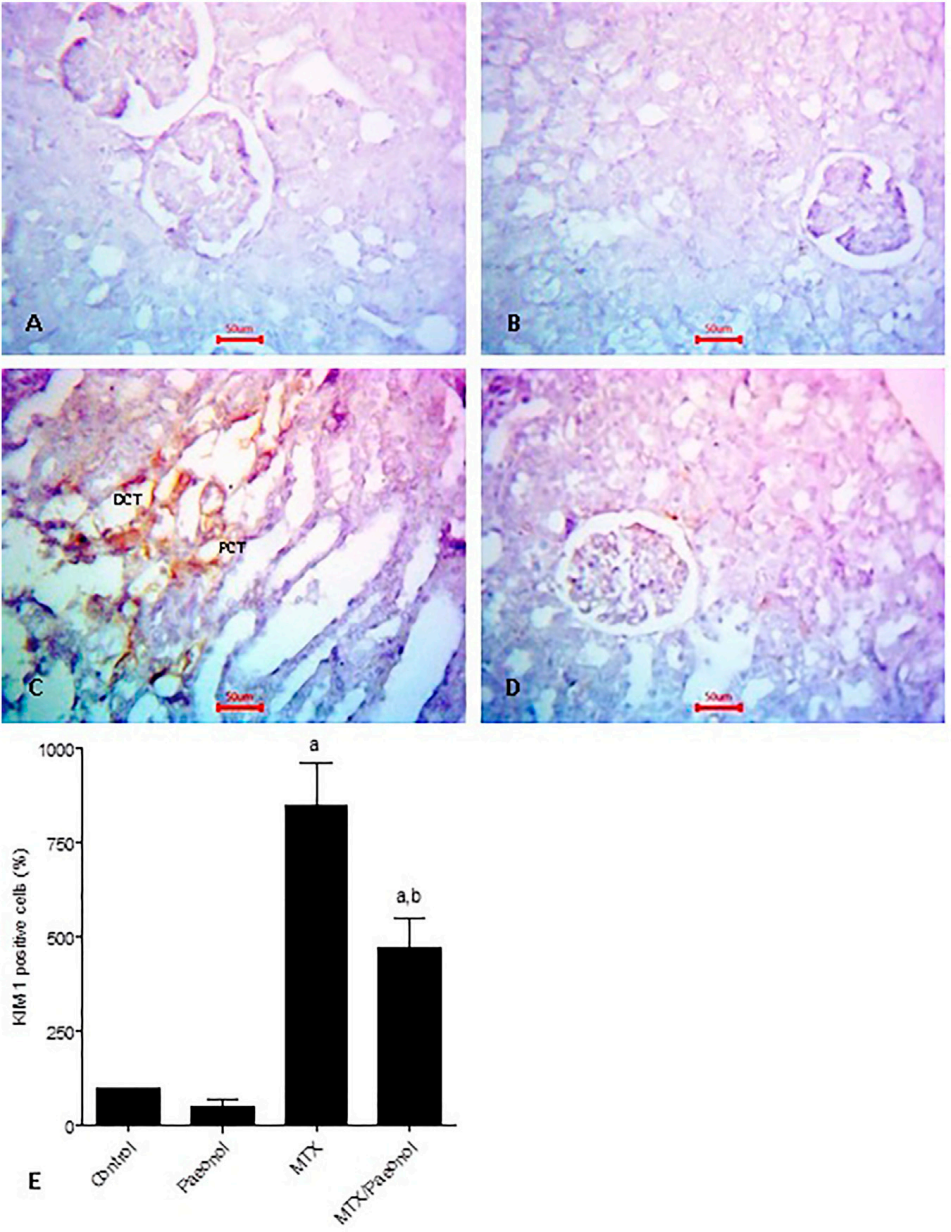
Kidney injury molecule-1 (KIM-1) immunohistochemical staining of the kidney after methotrexate (MTX) and/or paeonol administration. KIM-1 antibody–stained sections of the rat kidney (×400) from **(A)** control, **(B)** paeonol-treated, **(C)** MTX-treated, and **(D)** MTX/paeonol-treated rats. **(E)** Percent of positive cells compared to the control group, obtained from sections of each animal (*n* = 6 for each group), six fields/section, where results were considered significantly different when *p* < .05. ^a,b^ Significant difference compared to control or MTX groups, respectively.

To estimate the level of kidney injury, KIM-1 immunohistochemical expression was used, where both control and paeonol-treated groups had minimal KIM-1 expression (Figure 3A,B, respectively). MTX treatment, on the other hand, caused an apparent increase in KIM-1 nuclear expression (Figure 3C), which was decreased by combined treatment of paeonol with MTX compared to MTX alone (Figure 3D). This was statistically relevant as shown in the analysis of the percentage of KIM-1–positive cells (Figure 3E).

### Effect of Paeonol on Renal Inflammatory and Apoptotic Markers in MTX-Induced Toxicity

**FIGURE 4:**
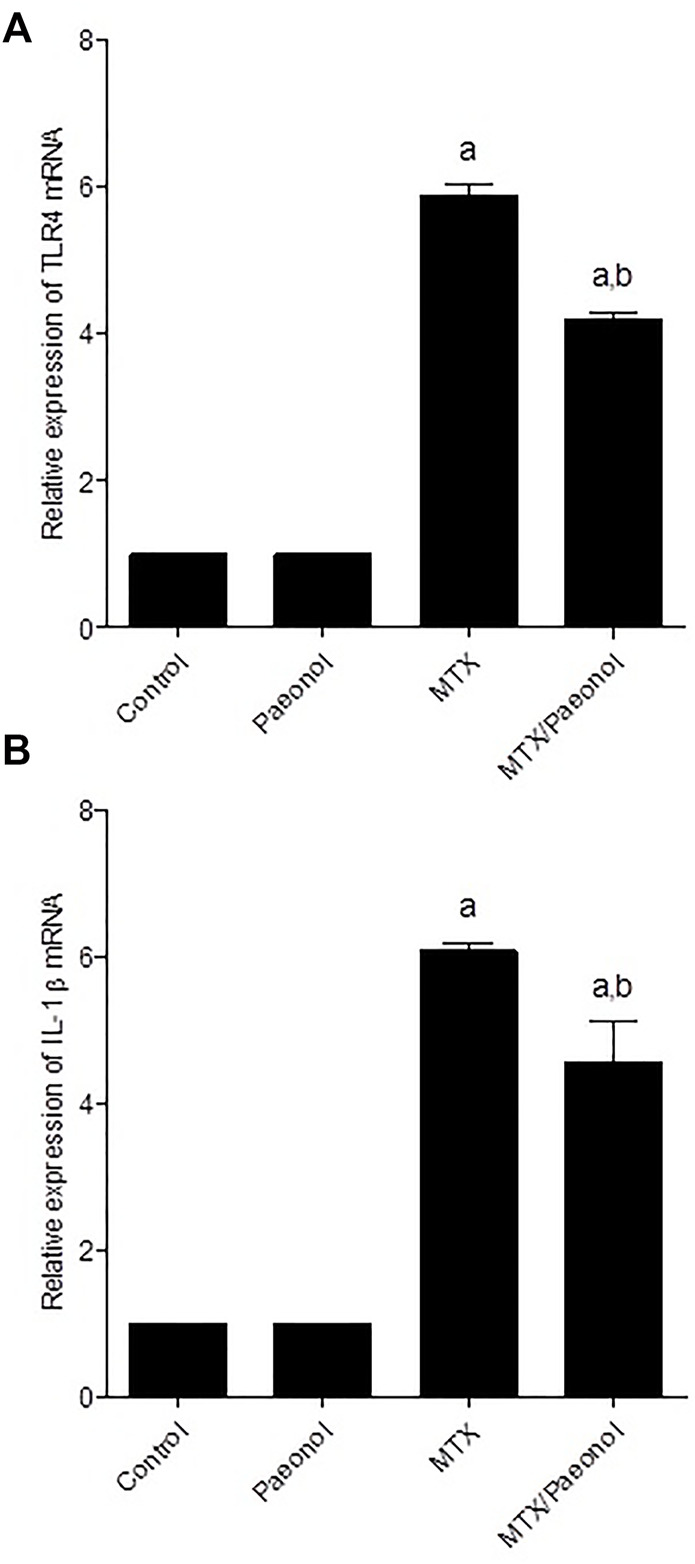
Renal toll-like receptor 4 (TLR4) and interleukin-1β (IL-1β) mRNA expression in the rat kidney after methotrexate (MTX) and/or paeonol administration. The mRNA expression of **(A)** TLR4 and **(B)** IL-1β was measured relative to that of glyceraldehyde 3-phosphate dehydrogenase (GAPDH). Results show means of six observations ± SEM. Values are considered significantly different when *p* < .05. ^a,b^ Significant difference compared to control or MTX groups, respectively.

**FIGURE 5:**
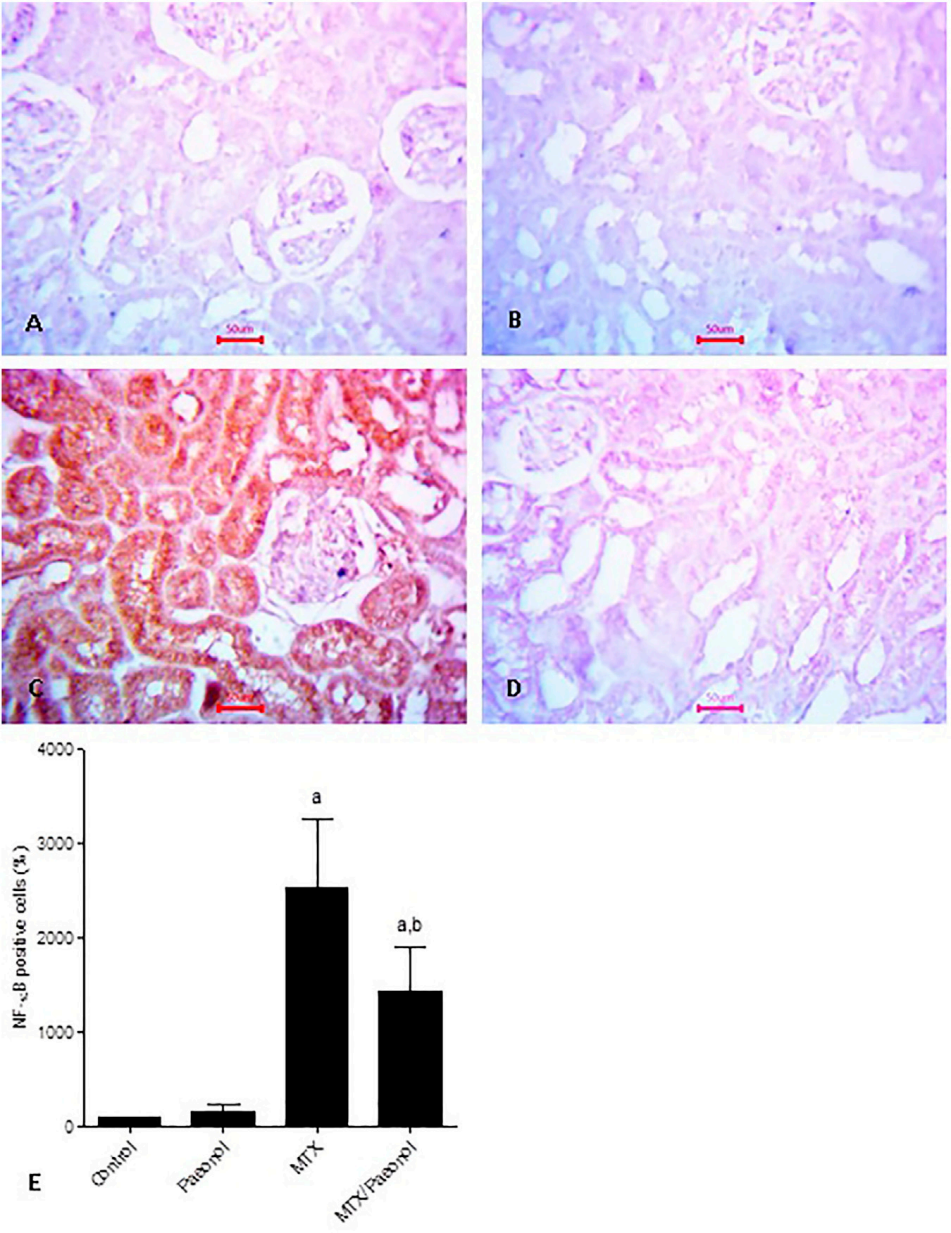
Nuclear factor-κB (NF-κB) immunohistochemical staining of the kidney after methotrexate (MTX) and/or paeonol administration. NF-κB antibody–stained sections of the rat kidney (×400) from **(A)** control, **(B)** paeonol-treated, **(C)** MTX-treated, and **(D)** MTX/paeonol-treated rats. **(E)** Percent of positive cells compared to the control group, obtained from sections of each animal (*n* = 6 for each group), six fields/section, where results were considered significantly different when *p* < .05. ^a,b^ Significant difference compared to control or MTX groups, respectively.

**FIGURE 6:**
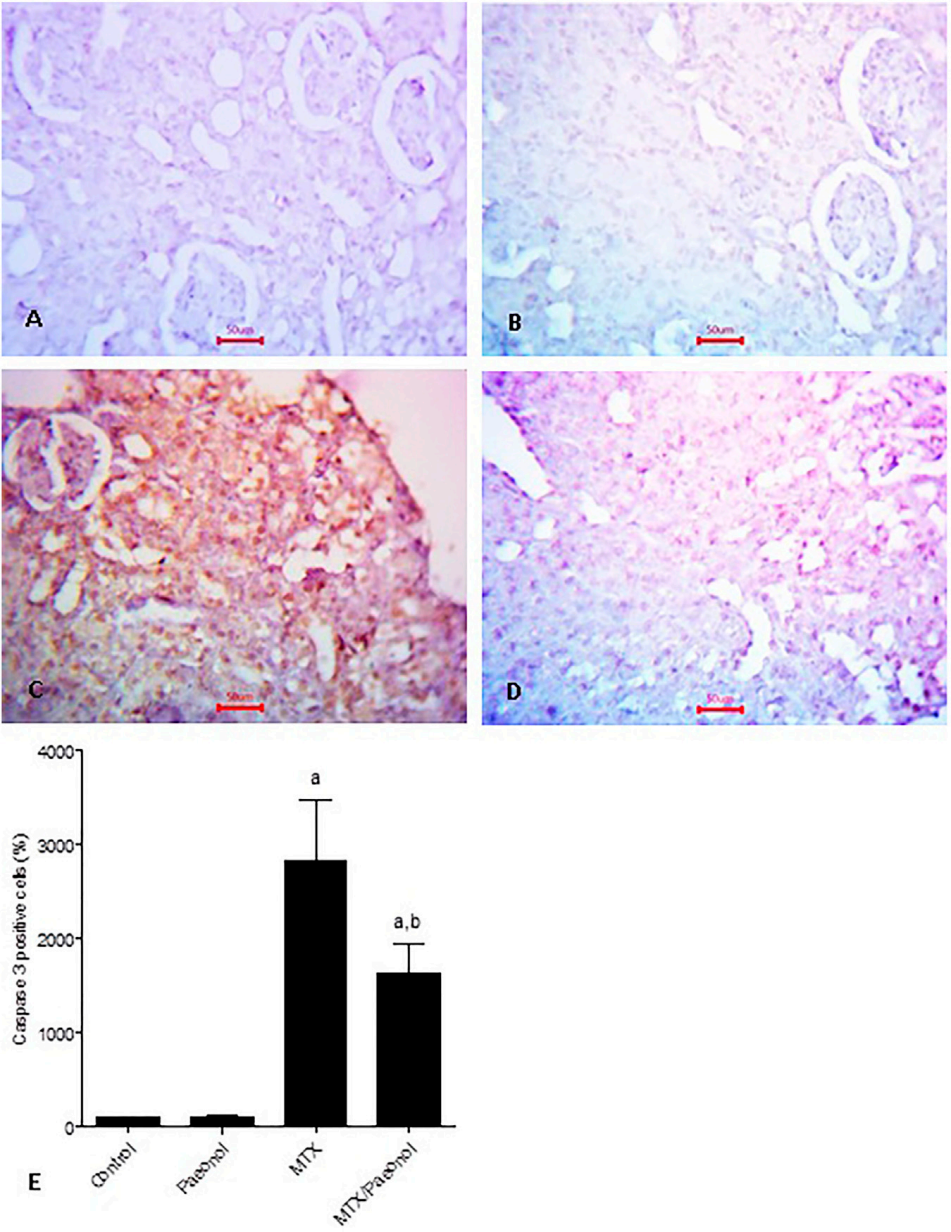
Caspase 3 immunohistochemical staining of the kidney after methotrexate (MTX) and/or paeonol administration. Caspase 3 antibody-stained sections of the rat kidney (×400) from **(A)** control, **(B)** paeonol-treated, **(C)** MTX-treated, and **(D)** MTX/paeonol-treated rats. **(E)** Percent of positive cells compared to the control group, obtained from sections of each animal (*n* = 6 for each group), six fields/section, where results were considered significantly different when *p* < .05. ^a,b^ Significant difference compared to control or MTX groups, respectively.

To estimate renal inflammatory status, TLR4 and IL-1β mRNA expressions (Figures 4A,B, respectively) as well as immunohistochemical expression of NF-κB (Figure 5) were examined. Paeonol treatment alone did not significantly affect any of the inflammatory markers tested. On the other hand, treatment with MTX alone significantly increased TLR4, NF-κB, and IL-1β compared to control. Co-administration of paeonol and MTX caused a significant decrease in the expression of all three markers compared to MTX alone. Using caspase 3 immunochemical staining as a marker for apoptosis, both control and paeonol-treated groups showed minimal expression of caspase 3 (Figures 6A,B, respectively). MTX treatment however significantly increased the expression of the apoptotic marker (Figure 6C) compared to control. Combining paeonol/MTX treatments caused a significant decrease in caspase 3 expression (Figure 6D) compared to the group receiving MTX alone. The significance of these results was reflected in the analysis of the percent of caspase 3–positive cells (Figure 6E).

### Effect of Paeonol on Renal Expression of P-gp in MTX-Induced Toxicity

**FIGURE 7:**
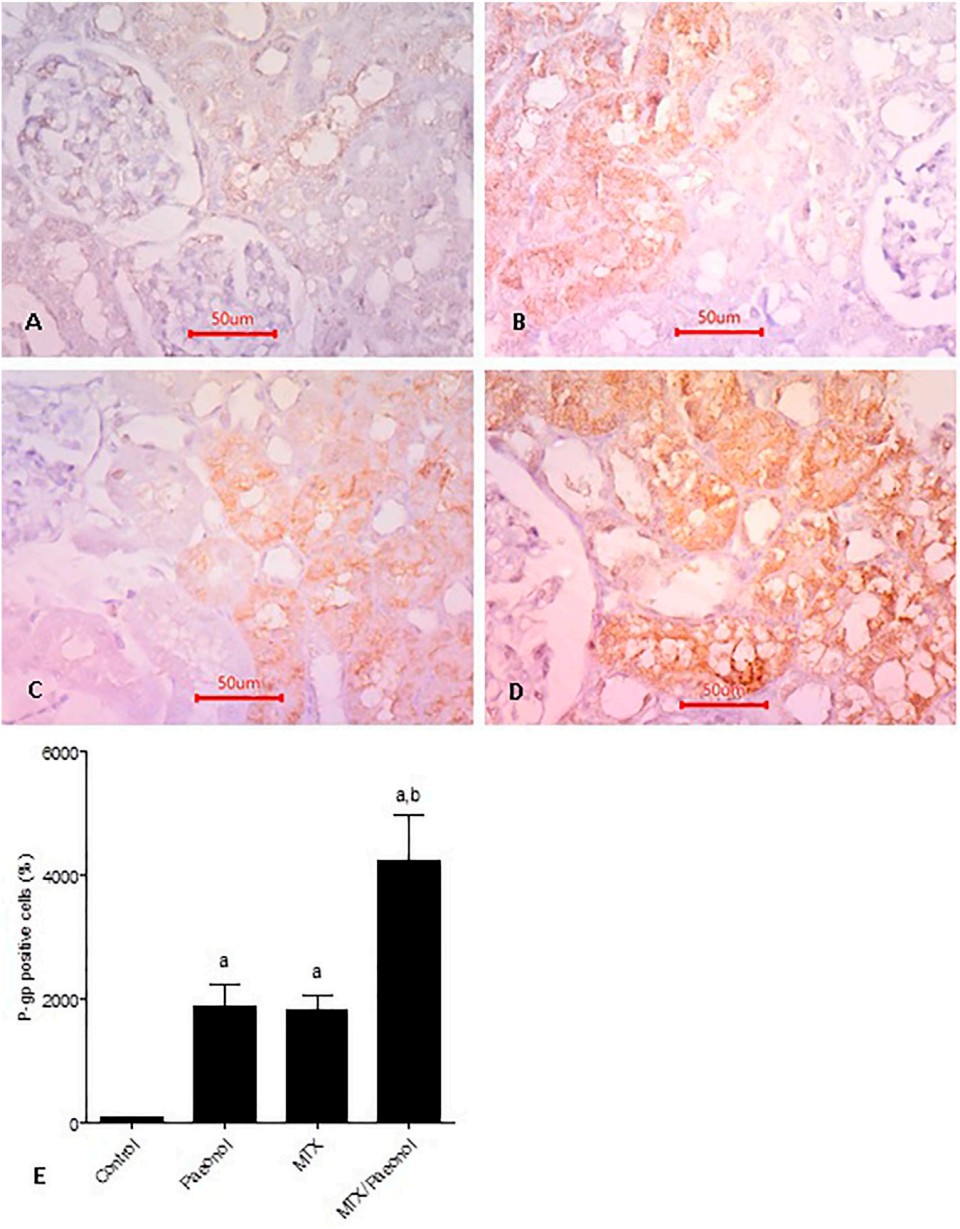
P-glycoprotein (P-gp) renal expression level in the rat kidney after methotrexate (MTX) and/or paeonol administration. P-gp antibody–stained sections of the rat kidney (×400) from **(A)** control, **(B)** paeonol-treated, **(C)** MTX-treated, and **(D)** MTX/paeonol-treated rats. **(E)** Percent of positive cells compared to the control group, obtained from sections of each animal (*n* = 6 for each group), six fields/section, where results were considered significantly different when *p* < .05. ^a,b^ Significant difference compared to control or MTX groups, respectively.

Basal expression of P-gp was shown in the control group (Figure 7A). Interestingly, both paeonol and MTX drastically increased renal expression of P-gp (Figures 7B,C, respectively) compared to control, and their combined administration caused further increase in expression of the efflux protein (Figure 7D). Setting control at 100%, it was shown that the percent of positive cells in paeonol- or MTX-treated groups was nearly 20 folds higher than control (Figure 7E). The effect of the paeonol/MTX combination was additive as it caused an increment of nearly 40 folds higher P-gp expression compared to control.

### *In Silico* P-gp Specificity of Paeonol

As shown in Table 2, paeonol shows a low probability for being a P-gp substrate and/or inhibitor with moderate reliability.

**TABLE 2:** *In Silico* P-glycoprotein (P-gp) specificity of paeonol.

Specificity	Classification	Probability	Reliability
P-gp inhibitor	Non-inhibitor	0.06	Moderate (0.72)
P-gp substrate	Non-substrate	0.09	Moderate (0.51)

### Effect of Paeonol on MTX Cytotoxicity in HCT-116 Colon Cancer Cells *In Vitro*

**FIGURE 8:**
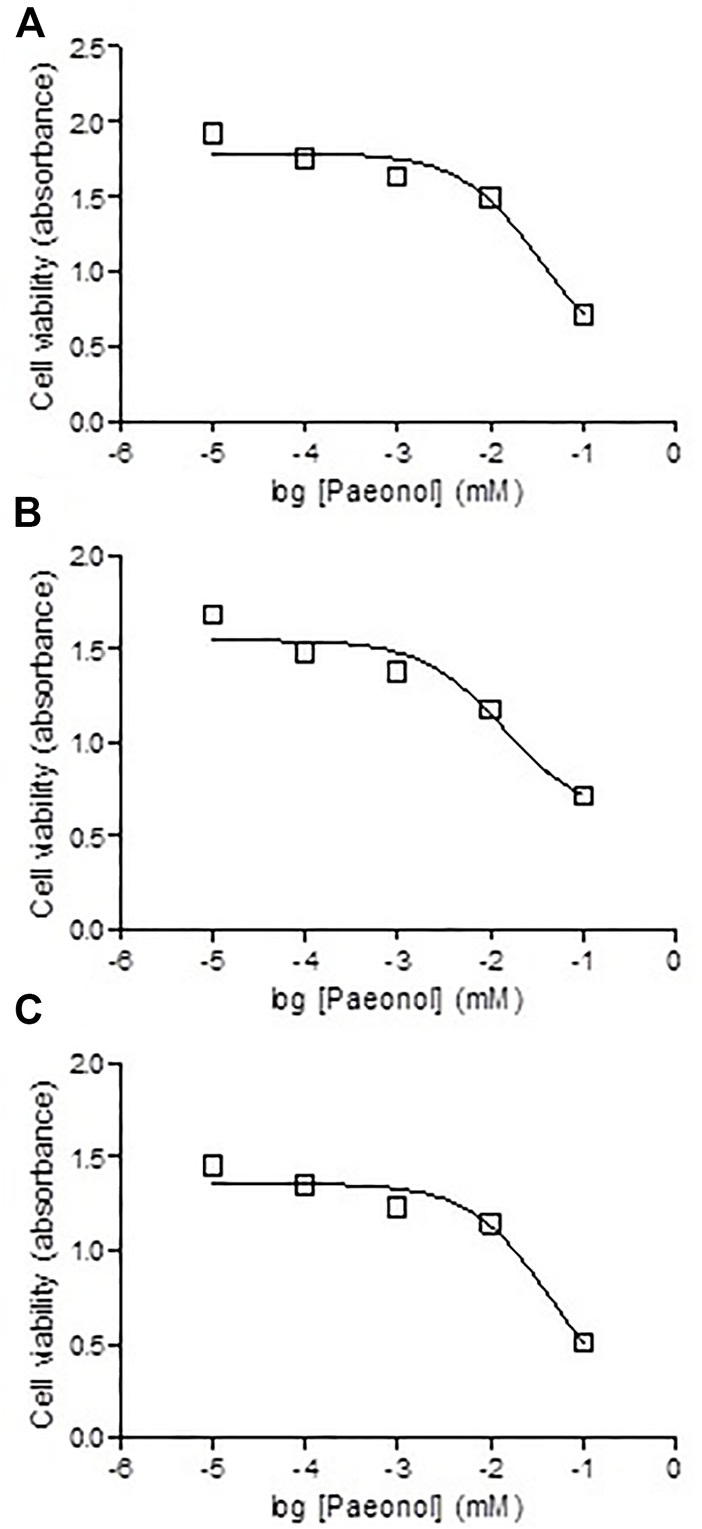
Effect of coadministration of paeonol and methotrexate (MTX) on cytotoxicity in colon cancer cells. Paeonol was administered to colon cancer cells in concentrations ranging from 0.01 to 100 μM concomitantly with MTX in concentrations of **(A)** 0.1, **(B)** 1, or **(C)** 10 μM.

To test the effect of administration of paeonol on MTX cytotoxicity, paeonol at concentrations ranging from 0.01 to 100 μM was administered to colon cancer cells together with MTX in a concentration either 0.1 (Figure 8A), 1 (Figure 8B), or 10 μM (Figure 8C). Interestingly, all tested paeonol concentrations when given in combination with MTX caused a dose-dependent progressive cytotoxic effect.

## Discussion

In the current study, we assessed the nephroprotective effect of paeonol against MTX-induced nephrotoxicity and found that paeonol improved kidney functions and structure and prevented MTX-induced renal toxic effects. We have previously reported that paeonol might have protective effects against MTX-induced cardiac and testicular toxicities (Al-Taher et al., 2020; Morsy et al., 2020a). Previous studies also showed that paeonol may confer nephroprotection against epirubicin and cisplatin in mice (Lee et al., 2013; Wu et al., 2017). Structurally, the kidney in the current study suffered from severe architectural disfigurement due to MTX treatment, which was nearly abolished by paeonol. Previous studies showed that paeonol could, after short- or long-term therapy of 7 days or 12 weeks, maintain renal structural integrity in unilateral ureteral occlusion or lead-stimulated kidney models, respectively (Liu et al., 2018; Zhou et al., 2019). As an indicator of kidney injury, level of renal KIM-1 was evaluated (Bonventre, 2009), and it showed an increase in the MTX-treated group, as expected (Aladaileh et al., 2019). Here, we show for the first time the effect of paeonol on KIM-1, as, when combined with MTX, paeonol caused a decrease in the renal KIM-1 level, providing proof of improvement of renal tubular injury induced by MTX.

It was previously shown that the mechanisms involved in MTX-induced renal toxicity may include oxidative stress, as manifested by a decrease in GSH and an increase in MDA and NO levels (Ibrahim et al., 2014; El-Sheikh et al., 2015; El-Sheikh et al., 2016). This was in line with the results of the current study. Few previous studies tested the protective effect of paeonol on the kidney (Liu et al., 2018; Mei et al., 2019; Zhou et al., 2019), but they focused on pathways other than oxidative stress, and, to the best of our knowledge, none of which investigated the effects of paeonol on oxidative stress in the kidney. Here, it was shown that paeonol improved all oxidative stress markers tested in kidney tissue. This was in line with the antioxidant properties exhibited by paeonol in other tissues such as the testis, stomach, and brain (Guo et al., 2020; Morsy et al., 2020a; Ghalami et al., 2021).

MTX, like other chemotherapeutic agents, may provoke systemic inflammation through TLR4 that activates NF-κB and enhances the production of inflammatory mediators such as IL-1β (Behranvand et al., 2021), which is in line with our current findings. Here, paeonol succeeded in improving the three inflammatory markers tested, provoked by MTX in the kidney. Previous studies showed that paeonol possesses nephroprotective effects against septic acute kidney injury (Mei et al., 2019) and endotoxin-induced acute renal damage (Fan et al., 2016), where both studies suggested that the mechanistic effect of paeonol was modulation of inflammatory markers, including TLR4, NF-κB, and IL-1β, which is in line with the current study.

Treatment with MTX in the present study caused upregulation of active executioner apoptotic marker caspase 3, similar to reported previous studies (Ibrahim et al., 2014; El-Sheikh et al., 2015; El-Sheikh et al., 2016). The present study also showed that paeonol succeeded in inhibiting apoptosis caused by MTX, as evident by the decrease in renal caspase 3 expression. This was in accordance with a single previous study that tested the effect of paeonol on caspase 3 renal expression in epirubicin-induced renal injury in mice (Wu et al., 2017). The complex cross talk between oxidative stress and inflammation on the one hand with apoptosis on the other hand was previously discussed (Taye and El-Sheikh, 2013), suggesting that paeonol might decrease the expression of caspase 3 secondary to the inhibition of oxidation and inflammation that might trigger the apoptotic process.

To investigate the role of P-gp as a nephroprotective mechanism of paeonol, in the current study, the possibility that paeonol is a substrate and/or inhibitor of P-gp was tested *in silico*. The results suggested that paeonol was neither, indicating that the mechanism does not involve acting on P-gp functionally. However, here, paeonol caused an increase in P-gp expression in the kidney. We also have previously shown that paeonol upregulated P-gp in testis (Morsy et al., 2020a), suggesting that P-gp is one of the mechanisms by which paeonol protects the sanctuary sites of the body. Interestingly, paeonol had the opposite effect on tumor cells as it was reported to downregulate P-gp in human breast cancer cells (Cai et al., 2014), emphasizing its adaptogenic property. Still, further studies are required to investigate the effect of paeonol/MTX combination on P-gp expression in different types of cancer cells and its implication on MTX anticancer efficacy. Here, MTX itself was also shown to upregulate renal P-gp. It is possible that such upregulation is a feedback protective mechanism, by which the kidney can increase the elimination of the compound assaulting it. Previous studies reported controversial results regarding the effect of MTX on P-gp expression as MTX downregulated P-gp in the liver (Mohamed et al., 2021), while upregulating it in fibroblast-like synoviocytes of rheumatoid arthritis patients (Qin et al., 2018), and it had no effect on the testis (Morsy et al., 2020a). These results may suggest that the MTX effect on P-gp expression is organ-specific. Previous studies also suggested that certain elements of the inflammatory pathway may modulate the expression of P-gp (Ho and Piquette-Miller, 2006), for example, TLR4 and NF-κB (Liu et al., 2015; Xie et al., 2017; Wang et al., 2020). In the current study, MTX upregulated, while paeonol/MTX downregulated the inflammatory markers TLR4 and NF-κB. But both compounds upregulated P-gp, suggesting that a different mechanism is involved other than the inflammatory pathway in upregulating renal P-gp.

The combined administration of paeonol with MTX showed progressive cytotoxic effects dose-dependently. The anticancer effect of paeonol was in line with previous studies conducted on cells from non–small-cell lung cancer (Zhang et al., 2020), colorectal cancer (Liu et al., 2020), ovarian cancer (Gao et al., 2019), and pancreatic cancer (Cheng et al., 2020). Still, the current study is the first to demonstrate the combined cytotoxic effect of paeonol and MTX.

## Data Availability

The original contributions presented in the study are included in the article; further inquiries can be directed to the corresponding author.
